# Aldehyded Dextran and **ε**-Poly(L-lysine) Hydrogel as Nonviral Gene Carrier

**DOI:** 10.1155/2013/634379

**Published:** 2013-08-21

**Authors:** Yumiko Togo, Katsu Takahashi, Kazuyuki Saito, Honoka Kiso, Boyen Huang, Hiroko Tsukamoto, Suong-Hyu Hyon, Kazuhisa Bessho

**Affiliations:** ^1^Department of Oral and Maxillofacial Surgery, Graduate School of Medicine, Kyoto University, 54 Kawahara-cho, Shogoin, Sakyo-ku, Kyoto 606-8507, Japan; ^2^Department of Paediatric Dentistry, School of Medicine and Dentistry, James Cook University, P.O. Box 6811, Cairns, QLD 4870, Australia; ^3^Center for Fiber and Textile Science Kyoto Institute of Technology, Creation Core Kyoto Mikuruma 213, 448-5 Kashii-cho, Kamigyo-ku, Kyoto 602-0841, Japan

## Abstract

*Background*. The expression term of the gene transfected in cells needs to belong enough inorder to make a gene therapy clinically effective. The controlled release of the transfected gene can be utilized. The new biodegradable hydrogel material created by 20 w/w% aldehyded dextran and 10 w/w% **ε**-poly(L-lysine) (ald-dex/PLL) was developed. We examined whether it could be as a nonviral carrier of the gene transfer. *Methods*. A plasmid (Lac-Z) was mixed with ald-dex/PLL. An *in vitro* study was performed to assess the expression of Lac-Z with X-gal stain after gene transfer into the cultured 293 cells and bone marrow cells. As a control group, PLL was used as a cationic polymer. *Results*. We confirmed that the transfection efficiency of the ald-dex/PLL had a higher transfection efficiency than PLL in 293 cells (plasmid of 2 **μ**g: ald-dex/PLL 1.1%, PLL 0.23%, plasmid of 16 **μ**g: ald-dex/PLL 1.23%, PLL 0.48%). In bone marrow cells, we confirmed the expression of Lac-Z by changing the quantity of aldehyded dextran. In the groups using ald-dextran of the quantity of 1/4 and 1/12 of PLL, their transfection efficiency was 0.43% and 0.41%, respectively. *Conclusions*. This study suggested a potential of using ald-dex/PLL as a non-carrier for gene transfer.

## 1. Introduction

Recently, many studies about gene therapy have been published. One of the achievements in gene therapy is safe and effective expression of the gene in the body. The naked DNA is the safe but rapidly degraded by nucleases, and it shows a poor cellular uptake. Success of gene therapy largely relies on gene delivery vectors [1, 2]. Although viral vectors have good transfer efficiency, the consequent immunogenic side effect is not negligible. On the other hand, advantages of safe nonviral carriersinclude an ability to introduce DNA into nondividing cells, avoidability of integration into the chromosome, lack of infective risks, and an expense potentially lower cost than viral vectors. However, nonviral carriers display poorer transfer efficiency than viral vectors [3]. Therefore, nonviral gene carriers such as cationic polymer [4], cationic lipid [5], and polysaccharide [6] have been developed to improve this weak point [1].

A cationic polymer and lipid form complexes with DNA by electrostatic interactions between positively charged amine of the polycations and negatively charged phosphate groups of the DNA. This can condense DNA into a relatively small size via ionic interactions, which is important for gene transfer because a small size is favorable for cellular uptake [4, 7]. In addition, the interaction between the complexes and negatively charged cell membranes can enhance DNA uptake by the cells [3]. These help to increase the transfection efficiency.

Recently, a wide range of materials has been investigated as a delivery vector of plasmid DNA. In the cationic polymer, poly(L-lysine) (PLL) is a well-known nonviral gene carrier that is biodegradable and nonantigenic. Nevertheless, it is toxic and tends to bind nonspecifically to the surface of all mammalian cells [8]. Therefore, it is relevant to modify PLL in order to be a safe and effective nonviral gene carrier [9]. Furthermore, the expression term of the gene transfected in a cell needs to be long enough in order to make a gene therapy clinically effective. The technique of controlled release of the transfected gene is utilized to lengthen the expression term by controlling the expression level and the term of gene. Until now, various controlled release techniques of gene have been reported [10–13]. Recently, a novel self-biodegradable bioadhesive hydrogel material has been developed. It was created by mixing aldehyded dextran and *ε*-PLL (ald-dex/PLL). A gel is formed after the mixture, which can control the degradation speed by changing the concentrations of acetic anhydride in PLL [14]. These characteristics are useful for controlled release of gene, since PLL can combine with DNA.

Of further note, mesenchymal stem cells (MSCs) have a high potential of proliferation and differentiation. The growth factors stimulating and inducing MSCs differentiation are indispensable for MSCs to differentiate into the desired cells [15]. Establishment of a gene transfer technique for MSCs enhances gene therapy and tissue engineering using MSCs [16]. In this study, we used the ald-dex/PLL to transfer the gene into MSCs and examined whether it can be used as a nonviral gene carrier of the gene transfer *in vitro*.

## 2. Materials and Methods

As a nonviral carrier of gene transfer, 10 w/w% PLL (Mw = 4,000) which contained 2 w/w% acetic anhydride and 20 w/w% ald-dex (Mw = 75 KDa) which was prepared by introducing the aldehyde group into dextran with an oxidizer were used.

A plasmid DNA named pCAGGS-lacZ which causes the cytoplasmic expression of *E. coli* *β*-galactosidase was used. The plasmid vectors were grown in *Escherichia coli* DH5 *α* and prepared with a Qiagen Plasmid Giga Kit (Qiagen GmbH, Hilden, Germany) according to the manufacturer's instructions. To verify the identity and purity of the plasmid vectors, agarose gel electrophoresis was performed after restriction endonuclease digestion. The plasmid DNA concentration was determined using a UV/visible spectrophotometer (DU-530, Beckman, Fullerton, CA, USA).

To assess the effect of transfection on mammalian cells, 293 cells obtained from the RIKEN Cell Bank (Tsukuba, Japan) and normal rat bone marrow cells obtained from the TAKARA BIO Japan were used. The cells were maintained in DMEM containing 5% fetal bovine serum (FBS) and penicillin as well as streptomycin (PS). Cell cultures were grown at 37°C in a humidified atmosphere of 95% air and 5% CO_2_. Upon confluence, 1.4 × 10^5^ cells per well were reseeded into 12 well plates and incubated for 24 hours.

The cases included 293 cells using the same amount of ald-dex and PLL mixed with plasmid DNA in DMEM, whilst the controls used only PLL with plasmid DNA in DMEM. The concentrations of plasmid DNA were 2 *μ*g and 16 *μ*g, respectively. Both were incubated at 25°C overnight. In the cultures of bone marrow cells, ald-dex of the amount of 1/1, 1/4, and 1/12 of PLL and PLL were mixed with plasmid DNA (concentration: 2 *μ*g) in DMEM and incubated at 25°C overnight.

After the cells were about 80% confluent, the medium was changed to DMEM supplemented with PS without FBS. The solution of the complexes was applied and incubated at 37°C in a humidified atmosphere of 95% air and 5% CO_2_ for 24 hours. In the cultures of 293 cells, only plasmids were applied as the controls. After 24 hours' transfection, the medium was changed to a new medium containing FBS. After another 24 hours, X-gal stain was performed to examine the transfection efficiency. The cells were fixed for 5 minutes in phosphate-buffered saline (PBS) containing 2% formaldehyde and 0.2% glutaraldehyde at the room temperature. They were subsequently washed with PBS and stained for 2 hours at 37°C in a 5-bromo-4-chloro-3-indolyl-*β*-D-galactopyranoside (X-gal) staining solution containing 1 mg/mL X-gal, 2 mM MgCl2, 5 mM, K3Fe (CN)6, and 5 mM K4Fe (CN)6·3H2O in PBS (pH 7.4). The experiments were repeated three times except the experiments using only PLL, which were repeated four times.

## 3. Results and Discussion

**Figure 1:**
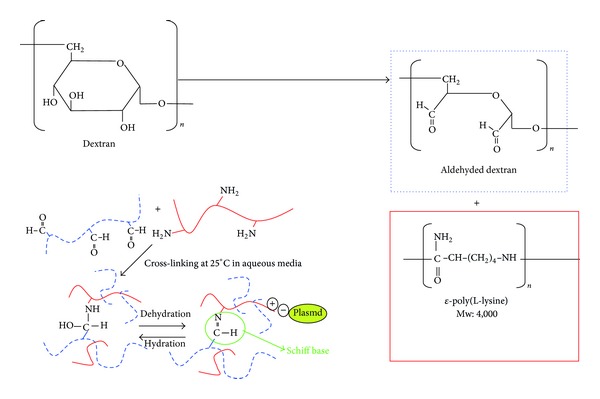
The chemical structure and cross-linking of the new biodegradable hydrogel composed of aldehyded dextran/PLL and plasmid DNA. After the mixture of the solution, gel formation proceeds on the basis of Schiff base formation. Plasmid DNA interacts with PLL by electrostatic interactions between positively charged amine of the PLL and negatively charged phosphate groups of the plasmid DNA.

Ald-dex/PLL hydrogel was formed by Schiff base formation between oxidized and aldehyded dextran and PLL [14]. The chemical structure and cross-linking are displayed in Figure 1. PLL has a sufficient number of primary amines with positive charges to interact with negatively charged phosphate groups of DNA. PLL/DNA complexes have a higher tendency to form precipitates [1, 17]. Previous studies reported that PLL has modified hydrophilic dextran with a reductive amination reaction between amino groups of PLL and reductive ends of dextran in order to increase the solubility of PLL/DNA complexes in aqueous media. The dextran chains do not disturb the electrostatic interaction between PLL and DNA [17]. The schema of ald-dex/PLL and plasmid DNA complex is shown in Figure 1. The complexes interact with negatively charged cell membranes and are taken into cells via endocytosis [1].

**Figure 2:**
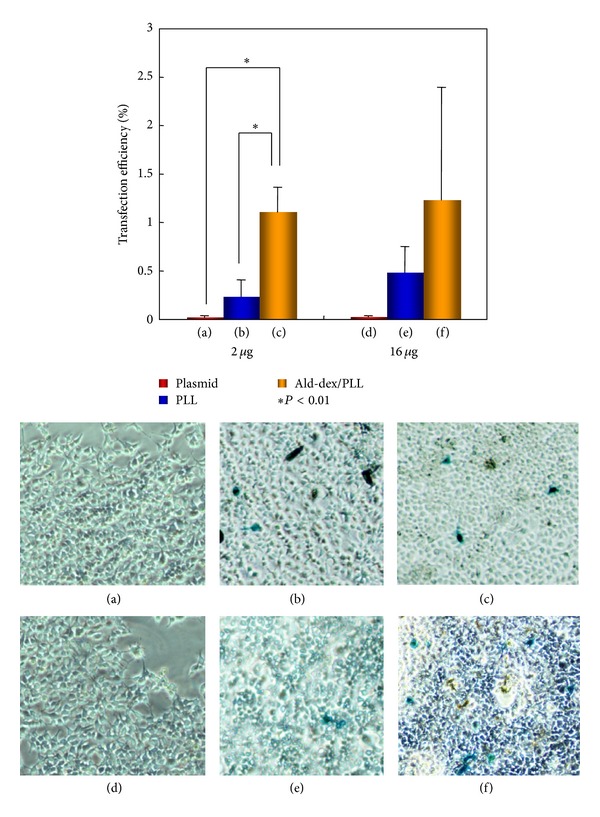
Gene transfection into 293 cells. (a), (b), and (c): results of gene transfection in plasmid of 2 *μ*g. (d), (e), and (f): results of gene transfection in plasmid of 16 *μ*g. (a) and (d) were carried out by only plasmid, and (b) and (e) were carried out by using PLL as a gene carrier, for the purpose of controls. (c) and (f) were carried out by using ald-dex/PLL as a gene carrier.

In the 293 cells, on the second day after lacZ gene transfer, we found that X-gal positive cells were present in all receiving transfections expect only plasmid transfection (Figure 2). In the groups with 2 *μ*g plasmids, transfection efficiency of PLL and ald-dex/PLL reached to 0.24 ± 0.17% and 1.10 ± 0.25%, respectively (*P* < 0.05 versus PLL). In the groups with 16 *μ*g plasmids, transfection efficiency of PLL and ald-dex/PLL reached to 0.48 ± 0.27% and 1.23 ± 1.16%, separately (*P* > 0.05). These demonstrated that ald-dex/PLL hydrogel could be used as a gene carrier. A higher transfection efficiency could be achieved by using ald-dex/PLL compared with using only PLL.

**Figure 3:**
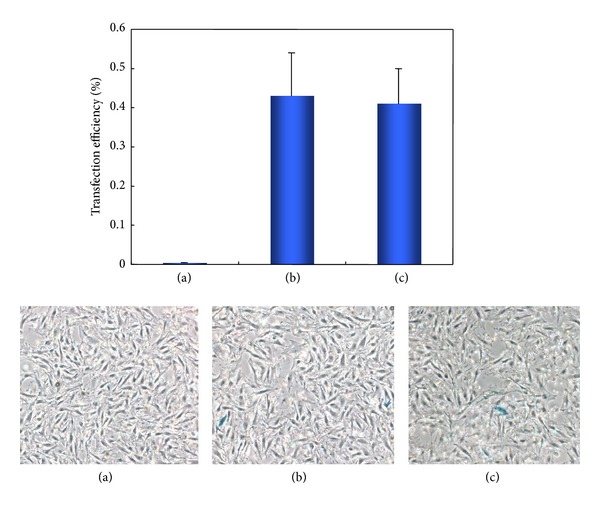
Gene transfection into MSCs. (a) Transfection with ald-dex (1/1 of PLL) and PLL. (b) Transfection with ald-dex (1/4 of PLL) and PLL. (c) Transfection with ald-dex (1/12 of PLL) and PLL.

Subsequently, we performed the gene transfer to MSCs existing in bone marrow cells. In the bone marrow cells, X-gal positive cells were not seen in the group using the same amount of ald-dex and PLL, whilst the specific cells were present in other groups (Figure 3). Transfection efficiency of the groups using ald-dex of 1/4 and 1/12 of PLL reached 0.43 ± 0.11% and 0.41 ± 0.09, respectively (*P* > 0.05). This study demonstrated the feasibility of transferring a gene into MSCs by decreasing the amount of ald-dex in ald-dex/PLL. An earlier study has suggested that the degree of grafting and length of graft chains had an influence on physicochemical properties of DNA in a complex. PLL-graft-dextran copolymers having a lower degree of grafting or shorter dextran chains could interact firmly with the DNAs and condense DNA [17]. This agreed with our findings. Furthermore, it has been reported that the PLL graft copolymers showing cell specific polysaccharide chains could perform cell specific delivery and express a foreign gene *in vivo* [18]. This study found different results between 293 cells and MSCs, since the uptake of complexes into cell is related to the cell specificity.

The novel self-biodegradable bioadhesive hydrogel, ald-dex/PLL, which could be degraded by hydrolysis in the body was developed. The favorable characteristics of ald-dex/PLL included a high bonding strength, a high flexibility, and a low cytotoxicity. Moreover, the onset time of hydrogel formation can be controlled by changing the amount of aldehyde introduced into dextran. The *in vitro* degradation speed of hydrogel can also be controlled by changing acetic anhydride concentration in the PLL solution [14].

A purpose of regeneration therapy is to induce the repair of defective tissues based on the natural healing potential in patients. It is desirable to use growth factors and/or genes to enhance cell proliferation and differentiation. The release technique is indispensable to make this possible. The success of controlled release of growth factors and genes relies on the incorporation of the growth factors and/or genes with appropriate carriers. Upon appropriate incorporation with carriers, the growth factors and/or genes are protected against proteolysis in the body, and consequently the active time can be lengthened [19].

This study has confirmed the feasibility of gene delivery using a novel self-biodegradable hydrogel. The ald-dex/PLL hydrogel showed a possibility to be used as a nonviral carrier for controlled release. Clinically, ald-dex/PLL combining plasmid DNA could be applied to the injured tissue to enhance tissue healing and regeneration. Thus, ald-dex/PLL combining BMP-2 encoding plasmids can be applied to a defect to enhance bone regeneration in case of fractures of a bone. Moreover, ald-dex/PLL hydrogel has been used as a surgical sealant [20]. If ald-dex/PLL combining with growth factor or genes is used as a surgical sealant, curing wounds with the new technique will become effective and feasible.

## 4. Conclusion

Our results suggest the potential of gene therapy using ald-dex/PLL hydrogel as a nonviral carrier. Furthermore, self-biodegradability and control of degradation speed in the ald-dex/PLL hydrogel indicated future applications in release technology.
